# Mitochondria: Regulators of Cell Death and Survival

**DOI:** 10.1100/tsw.2002.809

**Published:** 2002-06-11

**Authors:** David J. Granville, Roberta A. Gottlieb

**Affiliations:** The Scripps Research Institute, MEM-220, 10550 North Torrey Pines Road, La Jolla, CA 92037, USA

**Keywords:** mitochondria, apoptosis, caspase, cyt c, endonuclease, ceramide, Smac, DIABLO, cell death, protease, AIF, permeability transition, PARP, DFF, ICAD, EndoG

## Abstract

The past 5 years has seen an intense surge in research devoted toward understanding the critical role of mitochondria in the regulation of cell death. Apoptosis can be initiated by a wide array of stimuli, inducing multiple signaling pathways that, for the most part, converge at the mitochondrion. Although classically considered the powerhouses of the cell, it is now understood that mitochondria are also “gatekeepers” that ultimately determine the fate of the cell. The mitochondrial decision as to whether a cell lives or dies is complex, involving protein-protein interactions, ionic changes, reactive oxygen species, and other mechanisms that require further elucidation. Once the death process is initiated, mitochondria undergo conformational changes, resulting in the release of cytochrome c (cyt c), caspases, endonucleases, and other factors leading to the onset and execution of apoptosis. The present review attempts to outline the complex milieu of events regulating the mitochondrial commitment to and processes involved in the implementation of the executioner phase of apoptotic cell death.

## INTRODUCTION

Apoptosis has become the focus of intense scientific inquiry in recent years due to its pivotal role in physiological events, such as normal organ development; deletion of vestigial structures during embryogenesis; control of cell numbers; elimination of nonfunctional, harmful, abnormal, or misplaced cells; as well as its role in many genetic and acquired diseases[1]. Over the past 5 years, our understanding of the biochemical events regulating apoptosis has expanded dramatically. It is now apparent that mitochondria are the central regulators of cell death and survival. Although apoptosis can be initiated via a plethora of stimuli — including ultraviolet light, oxidative stress, viruses, chemicals, drugs, cytokines, and ligands — most pathways ultimately converge at the mitochondria, which then converts these signals into a proapoptotic response. Altered regulation of Bcl-2 and related proteins can significantly impair the ability of the mitochondrion to release the necessary signals to execute apoptosis. The present review details the current understanding as to how the mitochondrion deciphers proapoptotic signals and how it responds to these signals to initiate the final executioner stage of apoptosis.

## RECEPTOR-MEDIATED AND MITOCHONDRIAL PATHWAYS LEADING TO CASPASE ACTIVATION

Cysteinyl aspartate-specific proteases (also known as caspases) are believed to be key mediators of the apoptotic machinery. There are currently 14 homologs that have been identified in the caspase family. Caspases are present in the cytoplasm as zymogens, and their activation occurs by proteolytic processing by other proteases, often another caspase, or by autocatalysis to produce the active form[2]. Caspase activation is generally characterized by proteolytic processing between the small and large subunits followed by dimerization to generate the catalytically active heterotetramer. In the case of caspase-3, this is the primary mode of regulation. Caspase activation is generally recognized to be the "point of no return," wherein proteolytic cleavage of survival-signaling molecules and structural and DNA reparative proteins ensures the death of the cell.

Two major pathways to caspase activation are recognized: (1) receptor-mediated pathways and (2) mitochondria-mediated pathways. In the receptor-dependent pathway, death receptor occupancy (Fas, TNF-$\alpha$ receptor, TRAIL) leads to trimerization and recruitment of death adapter molecules, such as FADD and TRADD, to the cytoplasmic side of the membrane through "death domain" interactions. Molecules of procaspase 8 and/or 10 are recruited to the complex, where they undergo proximity-induced processing. The activated caspase-8 then processes downstream effector caspases, such as caspase-3, leading to the death of the cell[3].

In the second pathway, termed the mitochondrial pathway, cellular stress is relayed to mitochondria, which then respond by releasing a number of proteins from the intermembrane space into the cytosol (Fig. 1). The mitochondrial pathway to caspase activation is the general pathway involved in the cellular response to growth factor deprivation, DNA damage, cell cycle disturbances, and cytoskeletal perturbations[4]. The Bcl-2 family of pro- and antiapoptotic proteins are key regulators of the latter process. A number of Bcl-2 family members have been identified in mammals: Bcl-2, Bcl-$X_L$, A1/Bfl-1, Bcl-w, Nr13, and Mcl-1 serve to inhibit apoptosis, whereas Bax, Bik, Bak, Bad, Bid, Hrk, and Bcl-$X_S$ promote apoptosis[5]. The proapoptotic Bcl-2 family member translocates to the mitochondria, where it directs alterations in mitochondria, including the release of cyt c. Cyt c is a required cofactor for caspase-9 activation by Apaf-1, and formation of this complex is often referred to as the apoptosome. Activation of caspase-9 is followed by activation of executioner caspases-3, -6, and -7. The executioner caspases subsequently cleave many important cellular substrates, leading to events such as DNA fragmentation, nuclear membrane degradation, externalization of phosphatidylserine, and the formation of apoptotic bodies. Caspase-8 activation and Bid cleavage can also occur downstream of mitochondrial events, resulting in a feedback loop that enhances cyt c release[6,7,8,9]. The importance of this feedback mechanism has recently become evident with the discovery of the IAP (inhibitor of apoptosis) inhibitory protein Smac/DIABLO (*s*econd *m*itochondria-derived

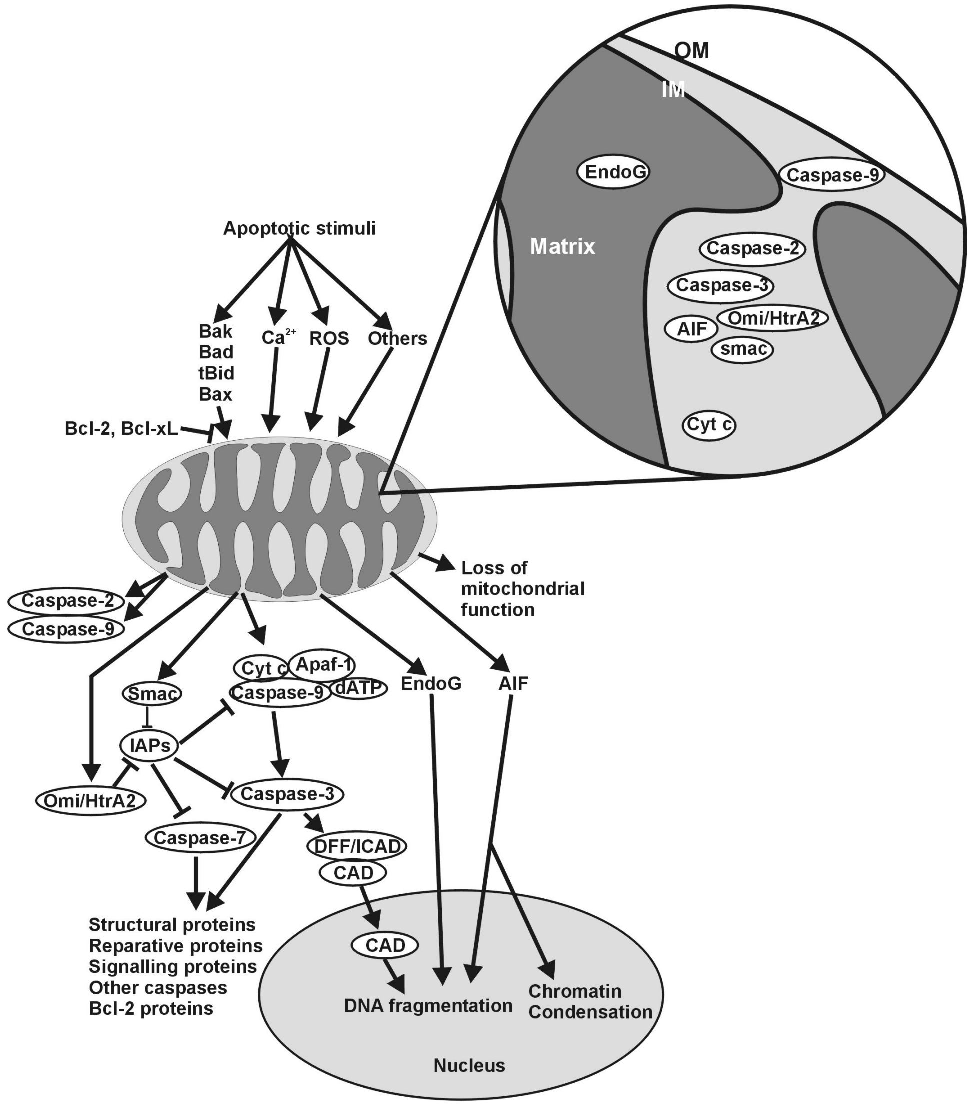

**FIGURE 1.** Mitochondrial pathways leading to cell death. A multitude of signals act upon mitochondria to elicit an apoptotic response. Mitochondria contain a number of proapoptotic factors in the outermembrane (caspase-9), the intermembrane space (caspase-2, -3, cyt c, Smac/DIABLO, Omi/HtrA2, AIF), and the matrix (EndoG). During apoptosis, cyt c is released into the cytosol and forms a complex with Apaf-1 and caspase-9 under dATP/ATP hydrolysis to form the apoptosome. Once activated, caspase-9 is involved in the activation of downstream executioner caspases (caspase-3, -6, -7). IAPs inhibit caspase activity and may target them for ubiquitous degradation. During apoptosis, Smac/DIABLO is released from mitochondria and inhibit the function of IAPs. The serine protease Omi/HtrA2 can also inhibit IAP activity and enhance caspase activity. Activated caspase-3 can induce DNA fragmentation via the cleavage of DFF/ICAD, thereby allowing CAD to translocate to the nucleus to fragment DNA. AIF is released and promotes large-scale DNA fragmentation and chromatin condensation. EndoG is another factor released from mitochondria that contributes to DNA fragmentation.

*a*ctivator of *c*aspase/*d*irect *IA*P *b*inding protein with *lo*w pI) that is released from mitochondria during apoptosis[10,11]. In brief, when released from mitochondria, cyt c binds to Apaf-1, triggering apoptosome formation and caspase activation. However, in the presence of high levels of IAP, caspase activation is inhibited. This inhibition may become permanent, as IAPs contain RING finger domains that can target caspases for proteosome degradation[12,13]. Such a system may provide the cell with a safety mechanism to avoid accidental cell death due to transient or incidental mitochondrial leakage of cyt c, a small molecule in comparison to Smac/DIABLO[14]. If mitochondrial damage is extensive, more Smac/DIABLO is released, along with more cyt c, thereby eliminating IAP inhibition and allowing caspase activation and apoptosis to ensue. Similar to cyt c, Smac release is inhibited by Bcl-2[15]. However, unlike cyt c, Smac release appears to be caspase dependent[15]. Thus, although not confirmed, Smac release may be dependent upon cyt c release and subsequent caspase activation. Further, based on these findings, Smac release may require a change in mitochondrial transmembrane potential ($\Delta\psi_m$), as this has been shown to be caspase dependent and downstream of cyt c release[16]. Whether Smac release is dependent on $\Delta\psi_m$ and whether its release is dependent on mitochondrial or cytosolic caspases require further elucidation.

Crosstalk between the receptor- and mitochondria-mediated apoptosis pathways occurs through caspase-8, which proteolytically transforms the BH3-only proapoptotic Bcl-2 family member, Bid, into a truncated form (tBid) capable of initiating cyt c release[17,18]. It should be noted that other proteases can also cleave and activate Bid; it therefore appears to be a sensor of intracellular protease activation[19,20,21]. The cleaved C-terminal fragment of Bid (tBid) translocates to the mitochondria, where it mediates cyt c release, possibly through inhibition of Bcl-2 function. Activation of the mitochondrial pathway by caspase-8/Bid allows amplification of the signal mediated by a small amount of caspase-8. This has led to a classification of cell lines as type I or type II, based on whether they can generate enough caspase-3 directly or whether they depend upon the Bcl-2-inhibitable step of mitochondrial amplification[22]. The resistance of Bid -/- mice to Fas-mediated hepatocyte death demonstrates the importance of Bid cleavage in receptor-mediated signaling[23].

Certain caspases have been shown to localize within mitochondria[24,25,26,27,28]. During apoptosis, caspases-2 and -9 are released into the cytosol[25,26]. Bcl-2 inhibits caspase release from mitochondria[25]. Furthermore, we have recently demonstrated differential processing of mitochondrial and cytosolic caspases and that Bcl-2 inhibits processing of both pools of caspases[28]. In Jurkat cells we found that caspases-2 and -3 localize to the mitochondrial intermembrane space, whereas caspase-9 is confined to the mitochondrial outer membrane and is exposed to the cytosolic face[28]. Interestingly, caspase processing appears to be stimulus dependent. For Fas-treated Jurkat cells, caspase activation takes place predominantly in the cytosol, whereas when these same cells are exposed to staurosporine, caspase activity is detected in both mitochondrial and cytosolic fractions[28]. In addition, caspase processing may also be cell-type–dependent. Mitochondria purified from different organs (liver, brain, kidney, spleen, and heart) contain both procaspase-9 and the processed, mature form of caspase-9[29]. However, the ratio of procaspase-9 to caspase-9 appears to be a cell-type–specific phenomenon. Surprisingly, the latter studies also demonstrated that mitochondria can contain a preprocessed caspase isoform in nonapoptotic cells[29]. During apoptosis, mitochondrial procaspase-9 translocates to the cytosol and to the nucleus[29]. Thus, although our understanding of caspases and their regulation has increased dramatically over the past 5 years, we have only just begun to realize their role in mitochondria, and further elucidation is required to solve this puzzle.

The role of Bcl-2 in the inhibition of cyt c release and caspase activation has been well documented[4,5]. However, an additional role for Bcl-2 is hinted at by the report that Bcl-2 interacts with carnitine palmitoyl transferase I (CPT I) in a yeast two-hybrid assay and in coprecipitation experiments[30]. Evidence for regulation of CPT I by Bcl-2 has not been published. But, given that carnitine inhibits caspases[31] and sphingomyelinase[32], regulation of carnitine and palmitoylcarnitine levels by CPT I, and indirectly by Bcl-2, could have a significant influence on the ability of the cell to undergo apoptosis. Interestingly, a mitochondrial sphingomyelinase was recently identified[33]. Whether carnitine inhibits this sphingomyelinase is unknown. The field has been largely focused on the events that follow cyt c release and has not considered the physiological consequences of mitochondrial alterations in apoptosis. Considerations of ATP synthesis, alterations in fatty acid metabolism, and free radical generation in the mitochondria have been addressed only marginally. We observe a dramatic shift in fatty acid metabolism during apoptosis with accumulation of the metabolite palmitoylcarnitine. The fact that the ratio of carnitine to palmitoylcarnitine can regulate caspase activity leads us to believe that alterations in lipid metabolism may play a coordinating role in apoptosis. In support of this concept, carnitine inhibits caspases while palmitoylcarnitine enhances caspase activity[31]. Furthermore, the ratio of carnitine to palmitoylcarnitine shifts dramatically during Fas-mediated apoptosis to favor caspase activation. Since Bcl-2 is reported to interact with CPT-I, the key enzyme controlling the balance of free to acylated carnitine, it raises the possibility that CPT-I represents a point of apoptosis control.

## MITOCHONDRIAL ARCHITECTURE AND MECHANISMS OF CYT C RELEASE

The topological organization of the mitochondrion raises concern about how cyt c release is accomplished. The mitochondrion is bound by an outer membrane that is perforated by the voltage-dependent anion channel (VDAC), which ordinarily permits free diffusion of small molecules (<1500 Da). Bcl-2 inserts into the outer membrane and projects out into the cytoplasm. In contrast to the limited surface area of the outer membrane, the inner mitochondrial membrane possesses a complex structure of deep tubular invaginations, or cristae, which join the circumferential inner membrane at narrow points called crista junctions[34]. The majority of the cyt c is associated with the inner membranes that form the cristae. The crista junctions are wide enough to accommodate cyt c, but could pose a barrier to rapid diffusion. Caspases -2, -3, and -9 are present and released along with cyt c, apoptosis-inducing factor (AIF), and Smac/Diablo.

Because of the pivotal role of cyt c in caspase activation, much effort has been directed at delineating the mechanism of its release from mitochondria during apoptosis. Bcl-2 and related antiapoptotic family members prevent its release[4]. Studies suggest that Bax translocates from cytosol to mitochondria[35,36], while others suggest that mitochondrial Bax undergoes a conformational change[37]. Members of the BH3-only family reside in the cytosol and, in response to a stimulus, translocate to the mitochondrial membrane, where they interact with Bax or Bak to achieve cyt c release. For instance, Bad is bound to protein 14-3-3 after phosphorylation. Dephosphorylation by protein phosphatase 1A or calcineurin permits its translocation to mitochondria[38,39]. Bid is a widely expressed member of the BH3-only subfamily and participates in receptor-mediated death signaling[17,18] as well as Granzyme B-mediated apoptosis[19,20], lysosomal leakage[40], and amplification of ultraviolet and cytotoxic death signaling[7,9]. Bid acts as a sensor of intracellular proteolysis and has been shown to be a substrate for caspases, Granzyme B, lysosomal proteases, and, in our own studies, calpain[21]. Cleavage of Bid in the random-coil region releases the N-terminal p7 fragment and exposes the BH3 domain of the p15 C-terminal portion of the molecule, referred to hereafter as truncated Bid or tBid, which translocates to the mitochondria and directs a conformational change in Bax or Bak that results in its insertion into the mitochondrial outer membrane[41,42]. Wei et al. have shown that tBid is unable to mediate cyt c release from mitochondria deficient in Bax and Bak[43]. Thus, cyt c release appears to require cross-talk between Bcl-2 homologs.

Contact points between inner and outer mitochondrial membranes are not located in proximity to crista junctions[34]. Recent immunogold electron microscopy studies localize tBid to these contact sites[44]. Additionally, using electron tomography, a profound reorganization of crista structure was shown to occur in response to tBid treatment, with expanded crista junctions[45]. Cyt c release may require a two-step process. Support for this notion has recently been shown by Ott et al.[46]. Cyt c is present as both loosely and tightly bound pools attached to the inner membrane by its association with cardiolipin. Dissociation of cyt c with cardiolipin involves breaching of the electrostatic and/or hydrophobic affiliations with cardiolipin, resulting in a soluble pool of cyt c. Once solubilized, permeabilization of the outer mitochondrial membrane by Bax is sufficient to allow the extrusion of cyt c into the cytocol. Furthermore, neither disrupting the interaction of cyt c with cardiolipin, nor permeabilizing the outer membrane with Bax alone is sufficient to trigger cyt c release[46]. Several other mechanisms of cyt c release have been proposed. In certain instances, the mitochondrial permeability transition pore opening results in matrix expansion followed by inner membrane unfolding. The secondary rupture of the outer membrane has been invoked as the mechanism for cyt c release[47]. Pore opening is associated with mitochondrial swelling as well as depolarization of the inner mitochondrial membrane. However, the observance of mitochondrial swelling is controversial and not consistently observed in response to all stimuli. For instance, studies of Bid- or Bax-treated mitochondria do not demonstrate significant swelling based on either light-scattering or ultrastructural analysis[16, 48]. Furthermore, it was shown that Bid and Bik induced cyt c release through a mechanism that did not involve VDAC or cause mitochondrial depolarization[49]. Evidence suggests that Bcl-2 family members regulate VDAC[50,51]. It has been proposed that Bcl-xL prevents closing of VDAC, which would lead to the buildup of ATP and creatine phosphate in the intermembrane space, with secondary mitochondrial dysfunction leading to the release of cyt c[52]. Another proposed mechanism for cyt c release is that Bax, alone or with other proteins, forms large channels. Shimizu et al. reported that Bax interacted with the VDAC of the mitochondrial outer membrane to form pores large enough to permit cyt c egress[53]. In related work, Bax has been reported to form large multimers that may create large pores in the outer membrane[54]. More recently, Pavlov et al. have described a channel, named mitochondrial apoptosis-induced channel (MAC), that contains Bax but does not require VDAC[55]. The other protein constituents of the channel remain to be elucidated, but the electrophysiologic properties of the channel suggest it may be large enough to accommodate cyt c and perhaps larger proteins. In summary, many proposals have been forwarded concerning cyt c release. Whether there is one common mechanism or whether its mode of release depends on the type of stimulus requires further studies.

## MITOCHONDRIA ARE THE CENTRAL COORDINATORS OF APOPTOSIS

Mitochondria play an essential role as the power plant of the cell, providing energy for specialized cell functions, as well as participating in calcium homeostasis, lipid metabolism, and uridine biosynthesis. Mitochondrial function is vital to cell proliferation and survival. However, mitochondria have a dark side: they sequester an arsenal of proapoptotic proteins, including cyt c, caspases, Smac/DIABLO, CIDE-B, and AIF. Furthermore, in studies using a proteomics/mass spectrometric approach to identify proteins released from isolated mitochondria during permeability transition, peptides from a total of 79 known proteins or genes were identified[56]. These proteins were involved in physiological functions ranging from mitochondrial protein import to benzodiazepine ligands to a number of catabolic enzymes. However, to date their roles in apoptosis are unknown. The serine protease Omi/HtrA2 is another factor that has been shown to be released by the tBid during Fas-mediated apoptosis[57]. Omi/HtrA2 has been shown to interact with XIAP, resulting in enhanced caspase activation[57].

Not all factors released from mitochondria are associated with caspase activation. AIF has been shown to be released from mitochondria in apoptotic cells and is responsible for caspase-independent large-scale DNA fragmentation and chromatin condensation[58]. Recently, another novel protein, Cell Death-Inducing DFF-45-like Effector (CIDE-B), has recently been shown by immunofluorescence to be localized in mitochondria[59]. Mitochondria also release endonuclease G, which ordinarily resides in the matrix[60,61]. How EndoG exits the matrix is unknown at present. Additional cellular stressors influence the outcome, including cytosolic calcium, intracellular pH, lipids (such as ceramide), redox poise, and signaling pathways including Raf-1[62] and Jun N-terminal kinase (JNK)[63].

Mitochondria, as the site of oxidative phosphorylation, are a potent source of free radicals. Whether free radical production increases or decreases after cyt c release is controversial[(15,64]. To the extent that free radical production may contribute to the death of the cell, mitochondria could certainly hasten the process. Mitochondria also play an important homeostatic function in calcium sequestration, damping peaks and then releasing the calcium slowly enough that it can be reaccumulated in the endoplasmic reticulum. Mitochondrial dysfunction leads to a gradual elevation of cytosolic calcium, initiating a series of enzymatic and transcriptional responses that may contribute to the demise of the cell. Bcl-2 has been shown to enhance mitochondrial homeostatic mechanisms to permit greater calcium sequestration[64].

## CONCLUSION

Mitochondria play a central role in the control of apoptosis. The outer surface of the mitochondrion is bristling with pro- and antiapoptotic members of the Bcl-2 family. Sequestered within the mitochondria are an increasing number of death-promoting factors that may be released as the outer mitochondrial membrane is disrupted. Members of the BH3 subfamily play an important role as cytoplasmic sensors of cellular injury, responding to proteolysis, phosphorylation, and disruption of the cytoskeleton. Understanding and delineation of the apoptosis-sensing and -executing machinery has already resulted in the clinical application of novel therapeutic compounds and will undoubtedly lead to many more discoveries as our knowledge of these pathways increases.
